# Therapeutic Notes

**Published:** 1896-08-22

**Authors:** 


### THERAPEUTIC NOTES.

### TUSSOL.

Tussol is one of the many derivates of antepyrine; it is in fact amygoalate or mandelate of antepyrine. It was originally introduced by Dr. Hinsberg, and has from time to time been recommended as a specific for whooping cough. It exists in the form of white granular crystals, soluble in water, and has a saline taste. In a paper recently published by C. Cattanio it is described as a valuable antespasmodic in cases of laryngitis, chorea and cerebral irritation. As an antepyretic it appears less active than antepyrine; it has no effect on the temperature when the latter is normal. No dangerous symptoms have been recorded as following its administration, and for children it seems a particularly useful drug for relieving those frequent reflex convulsions which arise from no very apparent cause of irritation. For whooping cough it may be given continuously in small doses from 1 to 5 grain according to the age of the child. The course of the disease is said to be greatly shortened by its administration.

Gaz. d'Osp., Ap. 11, 1896.

